# Inflammation causes tissue-specific depletion of vitamin B_6_

**DOI:** 10.1186/ar1821

**Published:** 2005-09-13

**Authors:** En-Pei Chiang, Donald E Smith, Jacob Selhub, Gerard Dallal, Yi-Cheng Wang, Ronenn Roubenoff

**Affiliations:** 1 Department of Food Science and Biotechnology, National Chung Hsing University, Taichung, Taiwan; 2 Comparative Biology Unit, Jean Mayer USDA Human Nutrition Research Center on Aging, Tufts University, Boston, MA, USA; 3 Vitamin Metabolism and Aging Laboratory (JS), New England Medical Center, Boston, MA, USA; 4 Biostatistics Unit (GD), New England Medical Center, Boston, MA, USA; 5 Nutrition, Exercise Physiology, and Sarcopenia Laboratory, New England Medical Center, Boston, MA, USA

## Abstract

Previously we observed strong and consistent associations between vitamin B_6 _status and several indicators of inflammation in patients with rheumatoid arthritis. Clinical indicators, including the disability score, the length of morning stiffness, and the degree of pain, and biochemical markers, including the erythrocyte sedimentation rate and C-reactive protein levels, were found to be inversely correlated with circulating vitamin B_6 _levels. Such strong associations imply that impaired vitamin B_6 _status in these patients results from inflammation. In the present study we examined whether inflammation directly alters vitamin B_6 _tissue contents and its excretion *in vivo*. A cross-sectional case-controlled human clinical trial was performed in parallel with experiments in an animal model of inflammation. Plasma and erythrocyte and pyridoxal 5'-phosphate concentrations, urinary 4-pyridoxic acid excretion, and the activity coefficient of erythrocyte aspartate aminotransferase were compared between patients and healthy subjects. Adjuvant arthritis was induced in rats for investigating hepatic and muscle contents as well as the urinary excretion of vitamin B_6 _during acute and chronic inflammation. Patients with rheumatoid arthritis had low plasma pyridoxal 5'-phosphate compared with healthy control subjects, but normal erythrocyte pyridoxal 5'-phosphate and urinary 4-pyridoxic acid excretion. Adjuvant arthritis in rats did not affect 4-pyridoxic acid excretion or muscle storage of pyridoxal 5'-phosphate, but it resulted in significantly lower pyridoxal 5'-phosphate levels in circulation and in liver during inflammation. Inflammation induced a tissue-specific depletion of vitamin B_6_. The low plasma pyridoxal 5'-phosphate levels seen in inflammation are unlikely to be due to insufficient intake or excessive vitamin B_6 _excretion. Possible causes of decreased levels of vitamin B_6 _are discussed.

## Introduction

Vitamin B_6 _deficiency results in adverse health consequences, including hyperhomocysteinemia [1] and possibly arteriosclerotic lesions [2]. We have reported that the degree of disease activity is associated with vitamin B_6 _indices in patients with rheumatoid arthritis [3,4]. Bates and colleagues reported suboptimal vitamin B_6 _status in inflammatory conditions and in the acute-phase response in the elderly population [5]. These observations have attracted attention partly because vitamin B_6 _deficiency and several markers of inflammation were both found to be independent risk factors for thrombosis [6,7]. Although several clinical trials and epidemiological studies have demonstrated the associations between vitamin B_6 _and inflammatory diseases, the association between vitamin B_6 _status and inflammatory markers has been contentious, and the cause-effect relationship between these two has not been elucidated.

Pyridoxine deficiency increased the degree of paw edema by 54% in a rat model of inflammation; it was therefore suggested that pyridoxine deficiency might enhance inflammation [8]. However, in healthy middle-aged adults, B vitamin status does not seem to be a strong correlate of circulating levels of inflammatory markers [9]. In contrast, a low level of circulating vitamin B_6 _was found to be associated with elevation of the inflammatory marker C-reactive protein independently of plasma homocysteine levels in the Framingham Heart Study cohort [10]. A recent study indicated that low plasma concentrations of pyridoxal 5'-phosphate are inversely related to major markers of inflammation and independently associated with increased coronary artery disease in the Italian population [11]. Decreased vitamin B_6 _status was also reported in patients after surgery and trauma [12]. In conditions with inflammation such as inflammatory bowel disease, low plasma levels of vitamin B_6 _are commonly found, especially in patients with active disease [13]. In a recent study we observed strong and consistent associations between vitamin B_6 _status and several indicators of inflammation in patients with rheumatoid arthritis [4]. Plasma pyridoxal 5'-phosphate was correlated with disease-related disability, morning stiffness, and degree of pain, C-reactive protein, serum albumin, and erythrocyte sedimentation rate [4].

The objective of the present study was to determine whether inflammation directly decreases the primary pools of vitamin B_6 _metabolism and storage, and to examine whether inflammation alters the excretion of vitamin B_6 _*in vivo*.

## Materials and methods

### Clinical trial

#### Study subjects

Thirty-three adults (aged at least 18 years) with rheumatoid arthritis were recruited from the Tufts University New England Medical Center Arthritis Center, Boston, as described previously [14]. Seventeen healthy control subjects, who did not differ in their age range or gender distribution from the subjects with rheumatoid arthritis, were recruited through advertisements in the greater Boston area. Study subjects were 18 to 80 years old. For the rheumatoid arthritis group, subjects had to fulfill the American College of Rheumatology criteria for rheumatoid arthritis [15]. The criteria for the classification of acute arthritis of rheumatoid include the following: (1) morning stiffness in and around the joints, lasting at least 1 hour before maximal improvement; (2) at least three joint areas simultaneously have had soft tissue swelling or fluid (not bony overgrowth alone) observed by a physician; (3) at least one area swollen of hand joints in a wrist, metacarpophalangeal, or proximal interphalangeal joint; (4) simultaneous involvement of the same joint areas (as defined in (2)) on both sides of the body; (5) rheumatoid nodules observed by a physician; (6) abnormal amounts of serum rheumatoid factor; (7) radiographic changes typical of rheumatoid arthritis on posteroanterior hand and wrist radiographs, which must include erosions or unequivocal bony decalcification localized in or most marked adjacent to the involved joints.

Written informed consent was obtained from all subjects in accordance with the regulations of the New England Medical Center/Tufts University Human Investigation Review Committee. Subjects with pregnancy, anemia (hemoglobin 10 mg/dL or lower), thrombocytopenia (platelet count 50,000/ μL or lower), abnormal serum hepatic transaminase (serum aspartate aminotransferase or alanine aminotransferase at least 50 IU/L), renal insufficiency (serum creatinine at least 1.5 mg/dL), diabetes, cancer or use of oral contraceptive were excluded. Thirteen of the 33 patients (39%) and 7 of the 17 controls (41%) were taking vitamin B6 or multivitamin supplements before enrollment. These subjects were asked to stop doing so for at least 2 months before their participation in the study.

#### Experimental protocol

This cross-sectional study was conducted in the New England Medical Center General Clinical Research Center. Before enrollment, each subject was examined by the study physician, and was screened by blood and urine analyses to ensure eligibility. Each subject was instructed to perform a 24-hour urine collection for measurement of 4-pyridoxic acid excretion. Patients taking methotrexate were asked to come in at least 24 hours after their weekly dose of the medicine to minimize any potential acute effect on laboratory outcomes. Urine specimens were kept at 4°C with no additive during the collection period. After completion of the 24-hour urine collection, subjects were asked to fast overnight for 12 hours. During the following morning, fasting blood was drawn into a tube containing EDTA for the determination of plasma pyridoxal 5'-phosphate, erythrocyte pyridoxal 5'-phosphate, erythrocyte aspartate aminotransferase activity coefficient, folate, and vitamin B_12_. Blood was also collected for hematology and chemistry analyses. Each patient's blood specimens were kept on ice and were centrifuged within 15 min of the blood draw.

### Animal model of inflammation

#### Animal

Thirty-six female 3-month-old Lewis rats were obtained from the National Institutes of Health. Animals were fed with the AIN93M diet during a 1-week washout period and during the experimental period. All animals were kept in individual mesh cages and acclimated to a 12-hour day/night cycle. The study protocol was approved by the Animal Care and Use Committee of National Chung Hsing University and of the Human Nutrition Research Center on Aging at Tufts University.

#### Induction of arthritis

After the washout period, animals of the same age and gender were sorted by body weight and assigned sequentially to the adjuvant arthritis or control groups (Table 1). Adjuvant arthritis was induced at baseline by injecting a single dose of *Mycobacterium butyricum *in mineral oil (complete Freund's adjuvant; 200 μL per rat) [16] into the base of the tail at the baseline time-point. The age-matched, paired-fed control animals received a saline injection.

#### Pair-feeding protocol

After the induction of adjuvant arthritis, for each rat in the adjuvant arthritis group one control rat, matched for age and weight, received the same amount of food on the next day. This protocol minimized variations in body weight and in vitamin B_6 _consumption due to different dietary intake.

#### Sample collection

Fasting blood samples were collected from the orbital sinus vein of each rat under anesthesia at baseline. Plasma was separated immediately by centrifugation and stored at -80°C until analysis. Sixteen animals (eight control and eight arthritic rats) were killed 21 days after the adjuvant injection, reflecting the condition of acute inflammation. The rest were killed on day 42, reflecting the condition of chronic inflammation. Animals were killed by thoracotomy and exsanguinations after anesthesia. Blood, liver, and skeletal muscle were collected and stored at -80°C until analysis for pyridoxal 5'-phosphate concentration. On selected days (days reflecting baseline, peak inflammation, and chronic inflammation), each animal was kept in an individual metabolic cage, specifically designed for the 24-hour collection of urine. The excretion of urinary 4-pyridoxic acid and creatinine was subsequently measured.

### Laboratory analyses

Blood hematology and chemistry analyses and urinalysis for human subjects were performed at the New England Medical Center Clinical Laboratory, Boston. For measurements of B vitamins, fasting blood was drawn from each human subject, and plasma was separated and stored at -80°C until analysis. Erythrocytes were washed three times with 0.9% saline and then an aliquot of packed erythrocytes was frozen before the measurement of erythrocyte aspartate aminotransferase [17]. The activity coefficient of erythrocyte aspartate aminotransferase was calculated by dividing pyridoxal 5'-phosphate-stimulated enzyme activity by the unstimulated activity. For pyridoxal 5'-phosphate analysis, the freshly washed erythrocytes were extracted with an equal volume of 10% (w/v) perchloroacetic acid. After centrifugation, the supernatants were stored at -70°C until analysis. Erythrocyte and plasma pyridoxal 5'-phosphate concentrations were assayed by a modification of the tyrosine decarboxylase enzymatic procedure of Camp and colleagues [18], in which a 20 μL aliquot of sample was precipitated with 80 μL of 5% (w/v) trichloroacetic acid for deproteinization. The erythrocyte pyridoxal 5'-phosphate results were expressed as nmol/L of packed erythrocytes at a hematocrit of 100%. The coefficient of variation (percentage of the mean) for the pyridoxal 5'-phosphate assay was 7.6% within assays and 5.7% between assays. Plasma folate, red blood cell folate, and plasma vitamin B_12 _were measured with Quantaphase II B_12_/Folate Radioassays (Bio-Rad; Hercules, CA). The weight of each 24-hour urine specimen was measured; aliquots were taken and stored at -20°C until analysis. No preservatives were added to the 4-pyridoxic acid or creatinine aliquots. 4-Pyridoxic acid was measured by high-performance liquid chromatography after urine had been mixed with an equal volume of 5% trichloroacetic acid for deproteinization [19]. The HPLC consisted of a Hitachi L-7100 intelligent pump connected to an L-2480 fluorometric detector. The ranges for plasma pyridoxal 5'-phosphate and urinary pyridoxic acid were in good agreement with published results. The range of erythrocyte pyridoxal 5'-phosphate concentrations in our subjects was similar to that in a previous study by Heiskanen and colleagues [20].

### Statistical analysis

Data were plotted so that they could be examined for normality before statistical analyses. Data for plasma pyridoxal 5'-phosphate and red blood cell folate levels in humans were log-transformed to achieve normal distributions, and the geometric means with the antilogarithm of the 95% confidence intervals are presented as results. Student's *t*-tests for independent samples were performed to determine whether there was a difference between patients with rheumatoid arthritis and controls in hematology measures, blood chemistry analyses, and vitamin profiles. For animal experiments, pooled Student's *t*-tests were performed to determine whether there was a difference between arthritic rats and matched control animals. All statistical analyses were performed with Systat 9.0 for Windows™ (SPSS, Chicago, IL).

## Results

### Demographic information

Thirty-six patients with rheumatoid arthritis were recruited from a previous study [14]. In the present study, a further 17 healthy subjects were recruited as control subjects. Three subjects from the patient group dropped out because of inconvenience of the 24-hour urine collection. Clinical and demographic characteristics of the study subjects are shown in Table 2. There were no differences in age, height, or weight between patients and controls, indicating that the control subjects and the patients did not differ in their physical conditions except for the presence of rheumatoid arthritis. The average disease duration in patients with rheumatoid arthritis was 10.8 ± 6.7 (mean ± SD) years. While participating in the study, 21 patients were taking non-steroidal anti-inflammatory drugs, 18 were taking prednisone, 16 were taking methotrexate, and 5 were taking gold. All patients had been taking the same medications for the 2 months preceding their entry into the study.

Patients and controls did not differ in the levels of serum albumin, creatinine, aspartate aminotransferase, and alanine aminotransferase. Patients had higher serum alkaline phosphatase levels, white blood cell counts and erythrocyte sedimentation rates than controls; patients also had a trend toward a reduced hematocrit, a smaller number of red blood cells and a lower hemoglobin level than the healthy control subjects (Table 2). Serum alkaline phosphatase concentration was inversely correlated with plasma pyridoxal 5'-phosphate concentration in all subjects (Pearson's correlation, *r *= -0.35, *P *= 0.012). In addition, serum albumin was modestly correlated with plasma pyridoxal 5'-phosphate in the patients (Pearson's correlation, *r *= 0.37, *P *= 0.04) but not in the control subjects.

### Vitamin B_6 _indices were altered in specific tissues during inflammation in humans with rheumatoid arthritis

In the human study, plasma pyridoxal 5'-phosphate concentrations were significantly lower in patients than in healthy subjects (with about 50% lower). This observation was comparable to our previous finding [3]. In contrast, no difference was found between patients and controls in erythrocyte pyridoxal 5'-phosphate or erythrocyte aspartate aminotransferase or 4-pyridoxic acid levels. No difference was found in concentrations of plasma folate, red blood cell folate or plasma vitamin B_12 _between patients and controls.

These results suggest that the lower vitamin B_6 _concentration in patients with rheumatoid arthritis is tissue-specific.

### Induction and progression of adjuvant arthritis in animals

Adjuvant arthritis was induced as described in the Materials and methods section. Arthritis onset was on day 14; the rats injected with adjuvant showed arthritic reactions including swollen paws and hind legs. Inflammation reached its peak on day 21 after the adjuvant injection. Joint swelling and body weight reduction continued for a further 4 weeks after the onset of arthritis (Table 1). Animals in the control and adjuvant groups were well matched in body weight at baseline before the induction of adjuvant arthritis.

### Adjuvant arthritis altered vitamin B_6 _contents in specific tissues in the Lewis rat model

At baseline before the adjuvant/saline injection, there was no difference between the adjuvant arthritis group and the saline-injected group in plasma concentrations of pyridoxal 5'-phosphate or urinary 4-pyridoxic acid excretion. These observations indicated that the animals were also well matched at baseline with regard to their vitamin B_6 _status. From then on, each control animal received the same amount of food as its experimental counterpart ingested during the previous 24 hours; this pair-feeding procedure minimized the impact of various vitamin intakes between the adjuvant-treated and the control animals. Adjuvant arthritis reached its peak 21 days after the injection. At peak inflammation, significantly lower levels of pyridoxal 5'-phosphate were found in the circulation and in liver in those arthritis rats, but muscle pyridoxal 5'-phosphate concentration seemed to be unaltered. A lower level of pyridoxal 5'-phosphate was also present in the circulation and in liver during chronic inflammation on day 42. However, prolonged inflammation did not alter the muscle content of pyridoxal 5'-phosphate. Plasma pyridoxal 5'-phosphate concentration was correlated with hepatic pyridoxal 5'-phosphate content during peak (Pearson's correlation, *r *= 0.51, *P *< 0.005) and chronic (*r *= 0.38, *P *< 0.04) inflammation.

### Adjuvant arthritis does not increase the urinary excretion of vitamin B_6 _at peak inflammation or during the chronic phase of inflammation

Despite the significantly lower pyridoxal 5'-phosphate levels in plasma and liver at acute inflammation, we did not observe a significant change in urinary 4-pyridoxic acid or creatinine excretion, indicating that the low level of vitamin B_6 _in plasma or liver at acute inflammation was not caused by excessive excretion of this vitamin. The 24-hour urinary excretion of 4-pyridoxic acid was highly correlated with the 24-hour urinary creatinine excretion throughout the study, indicating that renal function could be a significant determinant of 4-pyridoxic acid excretion in these animals. At baseline, 24-hour urinary 4-pyridoxic acid excretion was highly correlated with 24-hour urinary creatinine excretion in all rats (Pearson's correlation, *r *= 0.85, *P *< 0.0001). The 24-hour urinary excretion of 4-pyridoxic acid was correlated with 24-hour urinary creatinine excretion on day 21 (in control animals, *r *= 0.615, *P *= 0.005; in arthritic animals, *r *= 0.617, *P *= 0.014) and on day 42 (in control animals, *r *= 0.78, *P *< 0.0001; in arthritic animals, *r *= 0.80, *P *< 0.0001).

## Discussion

The results of these rat and human studies indicate that inflammation directly affects vitamin B_6 _metabolism differently in different tissues. Furthermore, the low vitamin B_6 _level is unlikely to be due to a decrease in food intake or the excessive excretion of vitamin B_6_. Adjuvant arthritis in Lewis rats is a useful animal model for studying vitamin B_6 _status during inflammation. Adjuvant arthritis decreased the pyridoxal 5'-phosphate pools in the circulation and liver, whereas it did not alter the pyridoxal 5'-phosphate pool in the skeletal muscle. Liver was studied because of its significant metabolic relevance and muscle was studied because it is the major store for vitamin B_6_. The lower plasma pyridoxal 5'-phosphate concentration in arthritic animals during inflammation was found to be in the physiological range seen in humans with rheumatoid arthritis. Despite the limited number of human subjects, the difference between patients and controls in plasma pyridoxal 5'-phosphate concentration was significant. The average plasma pyridoxal 5'-phosphate concentration in patients with rheumatoid arthritis was about 55% of the level seen in the healthy controls. The mean pyridoxal 5'-phosphate concentration in rats with adjuvant arthritis was about 53% of the controls at peak inflammation on day 21. Rat adjuvant arthritis reflected the altered plasma pyridoxal 5'-phosphate and it is a potential model for studying vitamin B_6 _status during inflammation.

Lumeng and colleagues suggested that plasma pyridoxal 5'-phosphate concentration reflects vitamin B_6 _status in the liver in healthy humans [21]. Kinetic studies in rats also indicate that changes in plasma pyridoxal 5'-phosphate content primarily reflect changes in the relatively small, but metabolically relevant and more rapidly exchanging, liver pool (as compared with muscle) [22]. However it was not clear whether this is true during inflammation. The reduced plasma pyridoxal 5'-phosphate level in our study implies that vitamin B_6 _status in the liver in these patients was altered, because we previously found good correlations between circulating pyridoxal 5'-phosphate level and vitamin B_6 _functional status measured by methionine load and tryptophan load in these patients [14]. Results from adjuvant arthritis were in agreement with this postulation. In the rat arthritis model, both plasma and hepatic pyridoxal 5'-phosphate concentrations were lower (Table 3). Furthermore, plasma pyridoxal 5'-phosphate concentration was correlated with hepatic pyridoxal 5'-phosphate content. These results suggest that the lower circulating pyridoxal 5'-phosphate levels observed in rheumatoid arthritis could reflect a decrease in hepatic pyridoxal 5'-phosphate pools, and plasma pyridoxal 5'-phosphate is a good indicator of liver B_6 _status during inflammation.

Our data imply that there are distinct metabolic roles for plasma and erythrocytes in vitamin B_6 _metabolism during inflammation, and that the impact of inflammation on vitamin B_6 _is tissue specific. In human subjects, despite the significantly lower pyridoxal 5'-phosphate in plasma (and possibly in liver), erythrocyte pyridoxal 5'-phosphate seemed to be adequate in patients with rheumatoid arthritis, because no difference was found between patients and healthy controls in the erythrocyte pyridoxal 5'-phosphate level or the activity coefficient of erythrocyte aspartate aminotransferase (Table 4). These observations are in agreement with the findings by Talwar and colleagues, which showed that pyridoxal 5'-phosphate decreases in plasma but not erythrocytes during systemic inflammatory response [23].

Data from our animal model imply localized vitamin B_6 _depletion during inflammation. Before the present study it was not clear how the pyridoxal 5'-phosphate pool in muscle might react to an inflammatory process. In rats with adjuvant arthritis, hepatic pyridoxal 5'-phosphate content was decreased, whereas muscle pyridoxal 5'-phosphate content remained unaltered, suggesting localized vitamin B_6 _deficiency during inflammation.

Skeletal muscle seems to be less sensitive to vitamin B_6 _deficiency in humans. In young healthy males receiving a defined diet restricted in vitamin B_6_, the muscle content of vitamin B_6 _is relatively resistant to vitamin B_6 _deficiency, whereas plasma pyridoxal 5'-phosphate is more sensitive to dietary vitamin B_6 _depletion [24]. We conclude that liver and muscle have distinctive roles as the body undergoes metabolic changes; skeletal muscle, the body's major storage site of vitamin B_6_, may turn over very slowly during inflammation.

### Low vitamin B_6 _status is unlikely to be due to lower intake or excessive excretion

Dietary intake is known to be a major determinant of vitamin B_6 _status. The arthritic and control rats showed decreases in plasma pyridoxal 5'-phosphate from the baseline levels. This was partly due to a decrease in overall food intake in both groups. However, the different vitamin B_6 _status observed between animals with adjuvant arthritis and control animals in the present study was not caused by different food intake between the two groups. Food intake of individual rats in the arthritis group was recorded daily, then a one-to-one match (each rat with adjuvant arthritis had its own weight-matched, saline-injected control) in food intake was arranged. This pair-feeding regimen in our animal experiments minimized the confounding effects of anorexia on the measures of vitamin B_6_.

Despite the significantly lower plasma pyridoxal 5'-phosphate in patients, 24-hour urinary 4-pyridoxic acid excretion in patients with rheumatoid arthritis did not differ from that of the healthy control subjects (Table 4). The low circulating pyridoxal 5'-phosphate level seen in these patients therefore did not result from excessive catabolism of vitamin B_6_. This is in agreement with the observation in our animal model. The 24-hour urinary excretion of 4-pyridoxic acid did not differ between control and rats with adjuvant arthritis, despite lower pyridoxal 5'-phosphate levels in plasma and liver in the adjuvant arthritic rats. To summarize these observations, the abnormal vitamin B_6 _status in rheumatoid arthritis results from the inflammatory process, and it is unlikely that it resulted from insufficient intake or excessive excretion of vitamin B_6_.

### Potential factors involved in the compartmentalization of pyridoxal 5'-phosphate during inflammation

In healthy populations, the variance in plasma pyridoxal 5'-phosphate can be explained to a great extent by vitamin intake, serum albumin, and alkaline phosphatase. The later two are physiological variables directly related to pyridoxal 5'-phosphate metabolism. In an elderly Dutch population it was reported that a combination of vitamin B_6 _intake, alkaline phosphatase, alcohol consumption, and albumin accounted for 30 to 40% variance in plasma pyridoxal 5'-phosphate [25]. Serum albumin is an acute-phase reactant that decreases during the flaring of active arthritis [26]. As the major protein for pyridoxal 5'-phosphate transport in the circulation, albumin might protect pyridoxal 5'-phosphate from hydrolysis [27]. In the present study, serum albumin was found to be correlated with plasma pyridoxal 5'-phosphate in patients whereas no such correlation was detected in the control subjects. Lower albumin levels in patients with more active arthritis may partly contribute to the lower pyridoxal 5'-phosphate level in these patients, although further study is needed for this postulation.

Many patients with arthritis have been reported to have elevated alkaline phosphatase [28], including those in the present study. Although still in the normal range, mean serum alkaline phosphatase levels in our patients were 26% elevated compared with healthy control subjects. Alkaline phosphatase hydrolyzes the phosphorylated form of vitamin B_6 _[29]; we therefore speculate that serum alkaline phosphatase could be another key determinant of the concentration of circulating vitamin B_6 _coenzyme during inflammation. Alkaline phosphatase has been shown to regulate extracellular levels of pyridoxal 5'-phosphate in humans [30,31], and abnormal vitamin B_6 _metabolism was found in alkaline phosphatase knock-out mice [32]. We found that the serum alkaline phosphatase level was inversely correlated with the plasma pyridoxal 5'-phosphate level in our subjects, which indirectly supports the above hypothesis. Compartmentalization of pyridoxal 5'-phosphate has been reported in the acute-phase response, such as the acute phase of myocardial infarction [33]. Because erythrocyte pyridoxal 5'-phosphate level seem to be normal whereas plasma and hepatic pyridoxal 5'-phosphate levels are significantly lower during inflammation, pyridoxal 5'-phosphate might be compartmentalized between tissues. The elevated alkaline phosphatase during inflammation may facilitate the mobilization and uptake of B_6 _vitamers, because vitamin B_6 _is taken up by tissues primarily in the form of pyridoxal.

In contrast, elevated serum alkaline phosphatase or reduced albumin did not provide a satisfactory explanation for the lower plasma pyridoxal 5'-phosphate level in rheumatoid arthritis, because the presence of disease remained a significant determinant of plasma pyridoxal 5'-phosphate concentrations after adjustment for serum alkaline phosphatase and albumin concentrations [34]. The low plasma pyridoxal 5'-phosphate level in patients with rheumatoid arthritis may also be attributed to elevated pyridoxal phosphatase activity during inflammation. It has been reported that the decrease in plasma pyridoxal 5'-phosphate characteristically seen in cirrhosis may be related to a substantial elevation of hepatic pyridoxal 5'-phosphate phosphatase activity [35]. McCarty hypothesized that the pro-inflammatory cytokine interleukin-6 might stimulate the activity of pyridoxal phosphatase in hepatocytes, in these patients, and the elevated enzyme may result in reduced plasma pyridoxal 5'-phosphate concentrations [36].

It remains uncertain whether the activity of pyridoxal 5'-phosphate phosphatase is altered in patients with arthritis, and this should be considered for future studies.

## Conclusion

A lower pyridoxal 5'-phosphate concentration in the circulation may reflect the removal of vitamin B_6 _coenzymes from the circulation to meet the higher demands of certain tissues during inflammation. In the animal model of adjuvant arthritis, lower pyridoxal 5'-phosphate levels in liver implied that it was largely hepatic pyridoxal 5'-phosphate that was used during inflammation. Further studies investigating the kinetics and regulation of B_6 _vitamers and enzymes in different body compartments are merited.

## Competing interests

The author(s) declare that they have no competing interests.

## Authors' contributions

All authors made substantive intellectual contributions to the present study. EPC conceived of the study, acquired partial funding, performed the human and animal experiments – including study designs, coordination, biochemical analyses, data acquisition, analysis, and interpretation – and drafted the manuscript. DES participated in the design and procedures of animal experiments. JS participated in the design of the study and the acquisition of funding, and was involved in revising the manuscript critically for important intellectual content. GED participated in the design of the study and performed the statistical analysis. YCW performed the animal experiments and analyses of metabolites. RR conceived of the study, acquired funding, and performed all clinical assessments in study subjects, and revised the manuscript critically for important intellectual content. All authors read and approved the final manuscript.

## Figures and Tables

**Table 1:** Changes in body weight in response to adjuvant arthritis in Lewis rats

Group	Body weight (g)		
	Baseline	Day 21	Day 42
Adjuvant arthritis	275.4 ± 85.3	228.9 ± 71.4 (-16.9%)	214.1 ± 60.7 (-22.3%)
Control	268.3 ± 86.3	259.1 ± 77.5 (-3.4%)	262.1 ± 73.0 (-2.3%)

Data are shown as means ± SD. Percentage changes in body weight at each time point (compared with baseline) are shown in parentheses. Day 21 represents the acute inflammation condition; day 42 represents the chronic inflammation condition.

**Table 2:** Characteristics of study subjects

Characteristic	RA (*n *= 33)	Controls (*n *= 17)	*P*
Sex (F:M)	23:10	10:7	-
Age	53.5 ± 12.4	52.9 ± 15.7	NS
Height (cm)	167.3 ± 11.0	170.1 ± 8.6	NS
Weight (kg)	77.1 ± 21.1	74.9 ± 17.9	NS
Rheumatoid factor (IU/mL)	89.4 ± 76.1	< 20	**<0.0001**
Chemistry			
Urinary creatinine (g/24 h)	1.0 ± 0.4	1.2 ± 0.3	NS
Serum urea nitrogen (mg/dL)	13.2 ± 4.3	15.6 ± 3.0	**<0.05**
Albumin (g/dL)	3.9 ± 0.4	4.0 ± 0.4	NS
Serum AST (IU/L)	19.4 ± 9.8	21.9 ± 11.2	NS
Serum ALT (IU/L)	21.4 ± 4.5	23.9 ± 7.5	NS
Alkaline phosphatase (IU/L)	72.2 ± 19.6	57.2 ± 14.3	**<0.01**
Hematology			
White blood cell counts (/nL)	7.3 ± 3.0	5.5 ± 1.2	**<0.01**
Hematocrit (%)	38.0 ± 3.5	39.8 ± 3.1	0.06
Hemoglobin (g/dL)	13.0 ± 1.6	13.7 ± 1.2	0.08
Mean corpuscular volume (fL)	88.3 ± 4.1	87.6 ± 6.4	NS
ESR (mm/h)	30.8 ± 20.4	8.2 ± 2.8	**<0.0001**

Data are presented as means ± SD. AST, aspartate aminotransferase; ALT, alanine aminotransferase; ESR, erythrocyte sedimentation rate; RA, rheumatoid arthritis. Bold *P *values are statistically significant.

**Table 3:** Effects of adjuvant arthritis on B vitamin status during inflammation in rats

Component	Adjuvant arthritis (*n *= 18)	Control (*n *= 20)	*P*
Baseline			
Plasma pyridoxal 5'-phosphate (nmol/L)	793.9 ± 191.3	744.2 ± 145.4	0.89
Urinary 4-pyridoxic acid (μg/d)	30.8 ± 17.3	30.0 ± 18.9	0.89
Acute inflammation			
Plasma pyridoxal 5'-phosphate (nmol/L)	252.0 ± 62.5	480.9 ± 144.0	**<0.0001**
Urinary 4-pyridoxic acid (μg/24 h)	27.7 ± 14.9	32.9 ± 17.2	0.249
Liver pyridoxal 5'-phosphate (nmol/g)	22.6 ± 1.7	27.4 ± 4.9	**0.035**
Skeletal muscle pyridoxal 5'-phosphate (nmol/g)	7.7 ± 1.8	7.2 ± 0.9	0.88
Chronic inflammation			
Plasma pyridoxal 5'-phosphate (nmol/L)	324.2 ± 91.6	393.7 ± 143.5	**0.033**
Urinary 4-pyridoxic acid (μg/24 h)	31.1 ± 11.8	27.1 ± 14.2	0.39
Liver pyridoxal 5'-phosphate (nmol/g)	21.1 ± 3.0	31.7 ± 4.7	**0.0001**
Muscle pyridoxal 5'-phosphate (nmol/g)	8.7 ± 4.8	6.4 ± 2.5	0.907

Data are presented as means ± SD. Day 21 represents the acute inflammation condition; day 42 represents the chronic inflammation condition. Bold *P *values are statistically significant.

**Table 4:** Indices of vitamin B status in patients with rheumatoid arthritis and control subjects

Component	Patients (*n *= 33)	Controls (*n *= 17)	*P*
Plasma pyridoxal 5'-phosphate (nmol/L)^a^	24.7 (19.5–31.1)	46.2 (35.3–60.3)	**0.001**
Red blood cell pyridoxal 5'-phosphate (nmol/L packed red blood cells)	39.7 (34.2–45.2)	33.1 (24.4–41.7)	0.182
α_EAST_	1.8 (1.8–1.9)	1.9 (1.8–2.1)	0.242
Urinary 4-pyridoxic acid (μg/day)^a^	0.8 (0.6–1.0)	1.1 (0.9–1.6)	0.08
Plasma folate (μg/L)^a^	10.2 (8.4–12.5)	9.5 (7.9–11.3)	0.185
Red blood cell folate (μg/L)^a^	302 (262–347)	274 (226–333)	0.404
Vitamin B_12 _(ng/L)^a^	434 (390–496)	398 (334–473)	0.371

Data are shown as geometric means and 95% confidence intervals. ^a^Data were log-transformed to achieve normality for statistical analysis. α_EAST_, erythrocyte aspartate aminotransferase activity coefficient. Bold *P *values are statistically significant.
